# The Roles of Hyaluronan/RHAMM/CD44 and Their Respective Interactions along the Insidious Pathways of Fibrosarcoma Progression

**DOI:** 10.1155/2013/929531

**Published:** 2013-09-05

**Authors:** Dragana Nikitovic, Katerina Kouvidi, Nikos K. Karamanos, George N. Tzanakakis

**Affiliations:** ^1^Department of Histology-Embryology, School of Medicine, University of Crete, 71003 Heraklion, Greece; ^2^Laboratory of Biochemistry, Department of Chemistry, University of Patras, 26110 Patras, Greece

## Abstract

Fibrosarcomas are rare malignant mesenchymal tumors originating from fibroblasts. Importantly, fibrosarcoma cells were shown to have a high content and turnover of extracellular matrix (ECM) components including hyaluronan (HA), proteoglycans, collagens, fibronectin, and laminin. ECMs are complicated structures that surround and support cells within tissues. During cancer progression, significant changes can be observed in the structural and mechanical properties of the ECM components. Importantly, hyaluronan deposition is usually higher in malignant tumors as compared to benign tissues, predicting tumor progression in some tumor types. Furthermore, activated stromal cells are able to produce tissue structure rich in hyaluronan in order to promote tumor growth. Key biological roles of HA result from its interactions with its specific CD44 and RHAMM (receptor for HA-mediated motility) cell-surface receptors. HA-receptor downstream signaling pathways regulate in turn cellular processes implicated in tumorigenesis. Growth factors, including PDGF-BB, TGF**β**2, and FGF-2, enhanced hyaluronan deposition to ECM and modulated HA-receptor expression in fibrosarcoma cells. Indeed, FGF-2 through upregulation of specific HAS isoforms and hyaluronan synthesis regulated secretion and net hyaluronan deposition to the fibrosarcoma pericellular matrix modulating these cells' migration capability. In this paper we discuss the involvement of hyaluronan/RHAMM/CD44 mediated signaling in the insidious pathways of fibrosarcoma progression.

## 1. Introduction

Cancer is a lethal disease characterized by uncontrolled cell growth, tumor formation, and loss of tissue organization. Primary tumors can be either caused by genetic alterations or by environmental factors. These alterations involve abnormalities in the regulation of basic cell functions, such as proliferation, differentiation, and apoptosis caused by genetic damage in oncogenes and tumor suppressor genes [1]. Within tumors, cancer cells often gain the ability to migrate, escaping from the normal mechanisms of control and thus invade surrounding tissues, leading to the formation of metastases via various tumor cell matrix interactions [2]. These interactions are considered continuous features of the metastatic cascade and play key roles in cell differentiation mechanisms.

Fibrosarcomas are rare malignant mesenchymal tumors originating from fibroblasts. The characteristic aspects of these tumors are the presence of immature proliferating fibroblasts or undifferentiated anaplastic spindle cells in a storiform pattern. Fibrosarcomas are usually localised in soft tissues, for example, muscles, connective tissues, blood vessels, and in lipid tissues [3]. Based on the presence and frequency of certain cellular and subcellular characteristics associated with malignant biological behaviour, sarcomas are also assigned a grade (low, intermediate, or high) [3]. Aetiology for sarcoma development has not been fully established; however, variations between ethnic groups in the incidence of rhabdomyosarcoma and fibrosarcoma, together with their occurrence in a number of heritable syndromes, suggest that genetic predisposition is important [4]. Comparative genomic hybridization further established the participation of genetic factors in sarcoma tumorigenesis [5]. Congenital fibrosarcoma is a paediatric spindle cell tumor of the soft tissues that usually presents before the age of 2 years. These tumors have a relatively good prognosis and only rarely metastasize even though they display histological features of malignancy and frequently recur. Therefore it is imperative to differentiate congenital fibrosarcoma from more aggressive spindle cell sarcomas that occur during childhood, particularly adult-type fibrosarcoma which can have an identical morphology but poor prognosis (Table 1) [6, 7]. Classic pathology defined any sarcoma with fibroblasts a fibrosarcoma, and as a result the diagnosis “fibrosarcoma” represented two-thirds or more of all sarcomas diagnoses. Due to improved methodology in tissue study, such as immunohistochemistry (testing of specific proteins within tumors) and cytogenetics (analysis of chromosomes), during the last 20 years the diagnosis of fibrosarcoma has become much rarer [8]. However, in spite of these methodological advances due to lack of positive diagnostic markers, fibrosarcoma is in some cases a diagnosis of exclusion, that is, once the possibility of other soft tissue tumors has been ruled out [9]. Mesenchymal tissues can also develop fibromas, benign tumors that are formed of fibrous or connective tissue. It is important to note that there is presently no specific “targeted” therapy against fibrosarcoma due to lack of identification of molecular targets [10]. 

The extracellular matrices (ECMs) are complicated structures that surround and support cells within tissues. Their main components are proteoglycans, fibrillar proteins including collagens, elastins, fibronectins and laminins as well as glycosaminoglycans (GAGs) [11–13]. Matrix proteins determine a varying degree of matrices organization. On the other hand, both bound GAG chains and free GAGs such as hyaluronan (HA) bestow voluminosity to the ECM, due to negative charges they carry and to their subsequent water binding ability. Cells interact with the ECM components not only through specific receptors, such as the integrin family members, but also through syndecans, CD44, and RHAMM receptors [14–16]. The attachment of these receptors to the ECM induces specific signal transduction pathways that lead to a variety of functional responses directing cellular organization of both the cytoskeleton and chromatin structures [17]. As a result the ECM participates in the regulation of almost all cellular functions and is thus indispensable for morphogenesis, tissue homeostasis, and different pathological processes [18–21]. 

It is noteworthy that the ECM provides a physical scaffold to which tumor cells attach and migrate and thus is required for key cellular events such as cell motility, adhesion, proliferation, invasion, and metastasis. The alterations of ECM components, cell shape, and changes at the cell-ECM interface are considered as important hallmarks of cancer [22–27]. Abnormal ECM also indirectly affects cancer cells by influencing the behaviour of stromal cells, including endothelial cells, immune cells, and fibroblasts, which are the main initial culprits that cause abnormal ECM production [28, 29]. Moreover, altered expression of ECM molecules also deregulates the behavior of stromal cells and promotes tumor-associated angiogenesis and inflammation, leading to generation of a tumorigenic microenvironment [25]. ECM remodelling and turnover are considerably increased most often due to modulation in the expression of degrading enzymes [30, 31]. Modification of the ECM can also be capable of reactivating dormant tumor cells, for example, mediated by integrin-FAK signaling [32]. As a result, abnormal ECM further perpetuates the local niche and promotes the formation of a tumorigenic microenvironment [33] and subsequent tumor metastasis. 

The above reports clearly show that the intrinsic structure of the tumor matrix has a key role on the insidious pathways of tumorigenesis. Importantly, fibrosarcoma cells were shown to have a high content and turnover of ECM components including hyaluronan, proteoglycans, collagens, fibronectin, and laminin [34–36]. In this paper we critically present and discuss how hyaluronan and its respective receptor for hyaluronan mediated motility (RHAMM) and CD44 receptors participate in the processes of fibrosarcoma tumorigenesis and dissemination. 

## 2. Hyaluronan Function and Synthesis

Hyaluronan is a high molecular weight (10^5^ to 10^7^ Da) GAG composed of alternating N-acetyl-glucosamine (GlcNAc) and glucuronic acid (GlcA) units [37]. It differs from the other members of the GAG family in that it neither contains sulfate groups nor is covalently attached to a core protein [38]. There are three different, but related, hyaluronan synthases (HAS), denominated HAS1, HAS2, and HAS3, that synthesize different hyaluronan sizes, with HAS1 and HAS2 producing high molecular weight HA (2000 kDa) [39]. The cleavage of hyaluronan on the other hand is performed by enzymes known as hyaluronidases (HYALs). The best studied mammalian HYALs are the HYAL1 and HYAL2. HYAL2 is located at the cell surface and cleaves the high molecular weight hyaluronan (HMWHA) into fragments of 20 kDa, whereas HYAL1 is intracellular and degrades the products of HYAL2 to small disaccharides [40].

Hyaluronan has remarkable physicochemical properties, such as the capacity to bind large amounts of water and form viscous gels, which are crucial in tissue homeostasis and biomechanical integrity. It also interacts with proteoglycans and other extracellular macromolecules forming a template that is important in the assembly of extracellular and pericellular matrices [41]. These properties bestow to hyaluronan the ability to act like a filter, allowing only small molecules to penetrate [42]. The extraordinary characteristics of hyaluronan have paved the way for its frequent use in tissue engineering [43].

The roles of hyaluronan *in vivo* are extremely heterogenous including the regulation of tissue repair, such as the activation of inflammatory cells in order to induce immune response [44–46] as well as the specific responses of epithelial cells and fibroblasts to injury [47–50]. Hyaluronan has also been correlated to pathological processes as high levels of hyaluronan on the surface of different cancer cells have been suggested to be connected with the pathophysiological conditions of cancer [51]. 

Importantly, the numerous biological functions of hyaluronan are size dependent. HMWHA (1,000 kDa) is present in intact tissues and is antiangiogenic as well as immunosuppressive, whereas low molecular hyaluronan (LMWHA) has been speculated to act as an endogenous signal for T-cell activation and has the ability to induce the processes of inflammation and angiogenesis [44, 52–54]. 

Certain biological roles of hyaluronan result from its interactions with a large number of HA-binding proteins, called hyaladherins [55–57]. Thus, hyaluronan binds to its specific cell-surface receptors, including CD44, RHAMM, and ICAM-1, to induce the transduction of a wide range of intracellular signals [58], which in turn regulate various cellular processes including morphogenesis, wound healing, and inflammation as well as being implicated in pathological conditions [57, 59–61].

## 3. Hyaluronan Expression in Tumor Cells and Its Role in Cancer Progression: Focus on Fibrosarcoma Cells

Importantly, hyaluronan deposition is usually higher in malignant tumors as compared to benign tissues [51, 62, 63], and in some tumor types the level of hyaluronan can predict tumor progression [62]. Furthermore, activated stromal cells adjacent to the cancer cells are able to produce a tissue structure rich in hyaluronan in order to promote tumour growth as well as to secrete factors that enhance cancer cell migration into the new matrix [64, 65]. Indeed, a striking difference in hyaluronan stromal expression was reported between the benign dermatofibroma and the malignant dermatofibrosarcoma protuberans. Thus, whereas the dermatofibroma specimens show just a faint hyaluronan staining of the tumor stroma, the dermatofibrosarcoma protuberans specimens exhibit high HA deposition [66]. 

The specific roles of hyaluronan metabolism in cancer cell function remain to be elucidated. In physiological *in vivo* systems it has been determined that hyaluronan synthesis is directly linked to the level of HAS mRNA [67–69]. Indeed, it has been suggested that the expression of HAS enzymes is the first and perhaps the most important determinant of the hyaluronan synthesis rate in a given cell type under specific circumstances [70]. Accordingly, HAS mRNA levels are known to influence the content of hyaluronan in fibrosarcoma [34, 36] and other mesenchymal type tumors [71]. Importantly, the expression profile and the activity of the HAS enzymes can stimulate tumor progression as has been shown in clinical studies on ovarian and colon carcinomas [72, 73]. Emerging data, however, strongly correlate the action of HA-degrading HYALs with the increase in the permeability of connective tissues and with the decrease in the viscosity of body fluids characteristic of various disease processes including cancer [74, 75]. Moreover, elevated extracellular levels of partially catabolized hyaluronan oligomers are found in certain malignancies [76]. 

It is noteworthy that in different tumor types there is a distinct regulation model for the expression of respective HAS and HYAL isoforms and their activities. Thus, it is indicative that the overexpression of HAS2 human fibrosarcoma HT1080 cells promoted anchorage-independent growth and tumorigenicity of the cells [77]. Furthermore, increased HAS1 and -2 expressions promoted migration abilities of these cells [34]. HYAL also seems to induce the tumor resistance of L929 fibrosarcoma cells to tumor necrosis factor and Fas cytotoxicity, in the presence of actinomycin D [78]. Interestingly intralesional injection of HYAL in a case of dermatofibrosarcoma protuberans was correlated to decreased margin width, and a postoperative wound size less than was expected [79, 80]. Moreover, decreasing HYAL-2 expression significantly attenuated migratory activity of HT1080 cells [34]. This emerging complex pattern as regarding HYAL expression and activity is corroborated with data obtained from other cancer tissues [81]. Thus, aggressiveness of human cancers including breast cancer [82], laryngeal cancer [83], tumors of the male genitourinary tract, and prostate [84] and urinary bladder cancers is correlated to increased HYAL1 expression [85]. In contrast an overexpression of HYAL1 suppressed tumorigenicity in a model for colon carcinoma [86]. 

These seeming contradictions of hyaluronan and respective fragments actions could be explained by taking into account the myriad of different hyaluronan molecular sizes. Studies have shown that the mass of the actual amount of the hyaluronan polymer determine its physiological function. Whereas, HMWHA is an established marker of intact, healthy tissues, the fragmented forms, which are indicators of distress signals and occur abundantly in tumors. Importantly, these fragments have been suggested to promote angiogenesis, stimulate production of inflammatory cytokines, and activate signaling pathways that are critical for cancer progression. LMWHA fragments may be truncated products of the synthetic reaction, the result of hyaluronidase activities [80] or degradation products of chemical reactions triggered by reactive oxygen species (ROS) [87]. 

Growth factors have been demonstrated to regulate the production of hyaluronan through the modulation of hyaluronan metabolic enzymes expressions under both pathological and physiological conditions [88]. This is also the case in fibrosarcoma cells, as FGF-2 stimulates in a cell-specific manner the migration capability of fibrosarcoma cells by decreasing HYAL-2 expression in HT1080 cells and by increasing HAS1 and -2 expressions [34]. In B6FS fibrosarcoma cells hyaluronan production was increased by TGFB2 and PDGF-BB actions [36]. 

Hyaluronan and derivatives can also support tumorigenesis by promoting tumor angiogenesis [89]. Firstly, hyaluronan accumulation in cancer tissues has been established to enhance the recruitment of monocytes and macrophages, which are important for angiogenesis [90, 91]. Secondly, hyaluronan seems to affect the binding ability of immunomodulatory cells. Thus, in inflamed colon tissues cell membranes were shown to form specific hyaluronan structures (cables) responsible for mediating leukocyte adhesion [92]. Thirdly, hyaluronan has been shown to maintain vascular integrity through endothelial glycocalyx modulation and caveolin-enriched microdomain regulation and interaction with endothelial hyaluronan binding proteins [93]. In vascular disease, also characterized by increased HYAL activity and ROS generation, HMWHA is broken down to LMWHA causing damage to the endothelial glycocalyx. Consecutively, LMWHA fragments can activate specific hyaluronan binding proteins to enhance actin cytoskeletal reorganization and inhibition of endothelial cell-cell contacts leading to decreased vascular integrity [94]. It is noteworthy that a decrease of vascular integrity is important both for tumor cell intravasation and tumor-associated angiogenesis. 

Interestingly, experimental evidence showed that the progression and vascularization of carcinomas may be dependent on the hyaluronan production by epithelial or stromal cells. It appears that in the absence of the stromal cells, respective tumors progressed more slowly because of their fewer stroma and lymphatic vessels content [94, 95]. Indeed, the important role of stroma-derived hyaluronan on tumor vascularization was demonstrated when the implantation of HAS2 null fibroblasts with epithelial tumor cells into nude mice resulted in attenuated tumor angiogenesis and lymphangiogenesis with impaired macrophage activation [96]. 

Hyaluronidases and HAS may also act as tumour suppressors or oncogenes [85, 97, 98]. These data strongly suggest that the definition of quantity as well as the quality of hyaluronan chains in tumors is fundamental for the regulation of cancer cell processes during the different stages of the metastatic cascade.

## 4. Receptor for Hyaluronan Mediated Motility (RHAMM)

RHAMM receptor was originally isolated from subconfluent fibroblasts in culture [99] and subsequently cloned from mesenchymal cells [100]. Various RHAMM isoforms are produced due to alternative splicing, and these transcript variants are suggested to be expressed in a specific cell type manner [101]. This receptor is unique among the hyaladherins due to its variable distribution on the cell surface, within the cytoplasm, in the nucleus, or secreted to the ECM [102, 103]. Namely, RHAMM belongs to a heterogeneous group of proteins that lack signal peptides and are traditionally predicted to be cytoplasmic proteins, but they also have a cell surface presentation by being GPI-anchored to the cell membrane [100, 104, 105]. The cell surface display of these proteins modifies the roles of tumor suppressors and promoters, and tumor cells commonly use this adaptive mechanism [15]. On the other hand, intracellular RHAMM binds to actin filaments, podosomes, the centrosome, microtubules and the mitotic spindle [58, 102, 106], thereby affecting crucial cellular processes in tumorigenesis [106]. Indeed, Telmar et al. [107] have recently proposed that intracellular RHAMM can bind directly to ERK1 to form complexes with ERK2, MEK1, and ERK1,2 substrates and suggested a model whereby RHAMM's function is as a scaffold protein, controlling activation and targeting of ERK1,2 to specific substrates [107]. Therefore, the function of RHAMM appears to be strictly linked to its expression and cellular localisation. 

Reports suggest that RHAMM expression is differentially regulated during the cell cycle and can be downregulated by the tumor suppressor p53 [108]. RHAMM protein expression during the cell cycle fits well into the picture proposed by several other studies that RHAMM binds to the mitotic spindle [109] and that, through interaction with HA, RHAMM affects microtubule spacing and stability [110]. These results underline the role of RHAMM as an important regulator of the cell cycle. 

RHAMM appears to be a key mediator of fibroblastoid cell functions. It has been proposed in fibroblasts that RHAMM targets and anchors MEK1/ERK1/2 to tubulin and that these MAPKs phosphorylate the tubulin-associated proteins that regulate microtubule dynamics [111]. The dynamic nature of microtubules has been linked to functions associated with cancer progression, including cell cycle progression and motility/invasion. Therefore, these results raise the possibility that microtubules are an important oncogenic target of transforming RHAMM protein forms, such as RHAMMΔ163, and are relevant targets of investigation in fibrosarcoma tumorigenesis. The importance of RHAMM for fibroblast motility is illustrated by a study which shows that RHAMM(−/−) fibroblasts fail to resurface scratch wounds >3 mm or invade HA-supplemented collagen gels in culture [112]. Furthermore, RHAMM is shown to be necessary for the localization of CD44 to the cell surface, formation of CD44-ERK1,2 complexes, and activation/subcellular targeting of ERK1,2 to the cell nucleus [112]. It was likewise shown that restricting cell surface RHAMM to the extracellular compartment by linking recombinant protein to beads, combined with expression of mutant active mitogen-activated kinase 1 (Mek1), rescued aberrant signalling through CD44-ERK1,2 complexes in resurface scratch wounds of RHAMM(−/−) fibroblasts. ERK1,2 activation and fibroblast migration/differentiation are also defective during repair of Rh(−/−) excisional skin wounds and results in aberrant granulation tissue *in vivo*. Therefore, Tolg et al. [112] identify RHAMM as an essential regulator of CD44-ERK1,2 fibroblast motogenic signaling required for wound repair. Moreover, a separate study demonstrated that native hyaluronan activated NF-*κ*B and activated protein 1, in fibroblasts during wound repair. Use of CD44 siRNA suggests that this hyaluronan receptor is partly implicated in the effects, although it does not rule out the involvement of other receptors including RHAMM [113].

## 5. The Role of RHAMM in Fibrosarcoma Tumorigenesis

Cell surface RHAMM is not highly expressed in normal tissues but is usually overexpressed in many advanced cancers [58, 102, 114]. The potential oncogenetic role of RHAMM is supported by various studies demonstrating an overexpression of RHAMM during tumor development and a prognostic significance of its expression in breast, colon, brain, prostate, endometrial, and pancreatic cancers, as well as in leukemia, aggressive fibromatosis, multiple myeloma, and melanoma [115–117].

**Figure 1:**
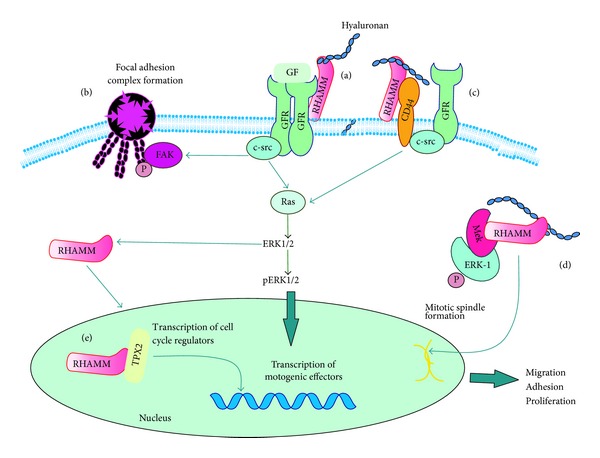
Hyaluronan/RHAMM/CD44-dependent signaling affects fibrosarcoma cell functions. (a) Interactions of cell membrane RHAMM with growth factor receptors (GFR) in a c-src/ERK1,2 dependent manner modulate transcription of motogenic effectors, and RHAMM/GFR interactions through c-src signaling induce FAK phosphorylation and focal adhesion complex formation. Interactions of cell membrane RHAMM with CD44 and GFRs in a c-src/ras/ERK1,2 dependent manner modulate transcription of motogenic effectors to regulate fibrosarcoma motility. (d) Cytoplasmatic RHAMM through RHAMM/MEK/ERK1,2 complex formation regulates mitotic spindle formation affecting cell growth. (e) Activated RHAMM positioned to nucleus forms complexes with transcription facors, for example, TPX2 to regulate expression of cell cycle mediators.

RHAMM/hyaluronan mediated signaling appears to be important in the process of fibrosarcoma tumorigenesis. Early studies have demonstrated that the overexpression of RHAMM in fibroblasts is transforming [100] and required for H-ras transformation [118], implying thus that RHAMM has a unique role in orchestrating events that are essential for transformation to occur. These events include the ability of RHAMM to alter focal adhesions in the cytoskeleton and elevate cell locomotion [118]. In an early report RHAMM/hyaluronan signaling was found to be obligatory for the stimulation of fibrosarcoma cell migration which is induced by transforming growth factor-beta 1 (TGF*β*1). Indeed, signaling is perpetrated through the formation of the RHAMM-HA complex because antibodies that inhibit RHAMM-HA binding simultaneously suppress TGF*β*1-induced increases in fibrosarcoma cell motility rate [119]. On the other hand, TGF*β*1 was found to stimulate multiple protein interactions at a unique cis-element in the 3′-untranslated region of RHAMM mRNA to stimulate its expression [120]. It was demonstrated that the treatment of fibrosarcoma (HT1080) cells with various molecular weight hyaluronan preparations resulted in regulation of their migration capacity in a manner strictly dependent on HA size [34]. In continuation, when the effects of hyaluronan on fibrosarcoma cell adhesion and the respective mechanism of its action were examined, it was demonstrated that HA regulates fibrosarcoma cell adhesion through interaction with its RHAMM receptor and consecutive activation of FAK and ERK1/2 signaling pathways (Figure 1) [121]. This is well explained by aprevious study reporting that in ras transformed fibroblasts, but not in the original cells, hyaluronan regulates cell motility via RHAMM by signaling transient protein-tyrosine phosphorylation within focal adhesions [122]. In this signaling pathway, FAK is transiently phosphorylated, followed by net dephosphorylation and focal adhesion turnover, which initiates cell locomotion [122]. Indeed, cells overexpressing RHAMM resemble ras-transformed fibroblasts and have elevated cell locomotion and focal adhesion loss, as well as tumorigenic and metastatic potential leading to fully metastatic fibrosarcoma [123]. Furthermore, it appears that RHAMM targets focal adhesions downstream of ras or via a parallel pathway that converges at the level of ras because expression of a dominant suppressor mutant of RHAMM was shown to reverse the transformation induced by ras and to stabilise focal adhesions [123]. Further, Hall and Turley (1995) proposed that tyrosine kinase pp60c-src is associated with RHAMM in cells and is required for RHAMM mediated cell motility. The established correlation between ras signaling and RHAMM-dependent mechanisms could be a key point in fibrosarcoma development in view of the previously reported correlation between specific ras mutations and the fibrosarcoma phenotype [124]. Specifically, the incidences of K-ras mutations have been described at a variable frequency in this tumor type, and an association has been reported between specific sarcoma types and mutations in codon 13 [125–127] and in codon 12 [128, 129]. Interestingly, K-ras 13-derived tumors were shown to resemble malignant fibrous histiocytomas (MFH), whereas K-ras 12-derived tumors were shown to resemble fibrosarcomas [128]. A further distinction has been reported in that the K12 tumors show differences in the expression or activation of other Ras downstream pathways, JNK, MAPK, AKT, Bcl-2, FAK, and cyclin B1, which could be correlated to their functional differences [124]. These studies highlight the importance of the ras-signaling pathways in mesenchymal tumors development.

RHAMM-HA binding is implicated during the process by which soluble RHAMM arrests ras transformed fibroblasts at G2/M without affecting their progression through S-phase [130]. Because RHAMM can regulate expression and regulation of cell cycle mediators, the reports demonstrating correlation between cell cycle mediator expression and fibrosarcoma development deserve due attention. In H-ras transformed fibroblasts soluble RHAMM induces mitotic arrest by suppressing Cdc2 and cyclin B1 expression [131]. Importantly, Oda et al. [132] demonstrated that variations of cell cycle regulators in related myxofibrosarcoma have specific prognostic implications. A comparison of conventional clinicopathological and immunohistochemical features and the assessment of the immunohistochemical expression of MIB-1, cyclin E, p21 and p27 may be helpful to distinguish low-grade myxofibrosarcoma (MFS) from low-grade fibromyxoid sarcoma (LGFMS) which have different metastatic properties [132]. Microarray analysis identified a novel set of AP-1 target genes, including the tumor suppressor TSCL-1, and regulators of actin cytoskeletal dynamics, including the gelsolin-like actin capping protein CapG. The demonstration that AP-1 regulates the expression of genes involved in tumor cell motility and cytoskeletal dynamics in a clinically derived HT1080 human tumor cell line identifies new pathways of control for tumor cell motility [133]. Skp2 and cyclin-dependent kinase subunit 1 (Cks1) are involved in posttranscriptional degradation of p27 (Kip1) tumor suppressor. The prognostic utility of p27 (Kip1) and its interacting cell cycle regulators in myxofibrosarcomas were analyzed: Skp2 overexpression is highly representative of the biological aggressiveness of myxofibrosarcomas and plays an important prognostic role [134]. The participation of RHAMM in the development of fibromatosis, the aggressive mesenchymal tumor, has also been demonstrated [135]. Furthermore, it is reported that RHAMM regulates proliferation of cells with sparse cell-cell contacts, indicating RHAMM blockade as a potential therapeutic target for this otherwise difficult-to-treat neoplasm [135]. Likewise, the overexpression of RHAMM in osteoblastic MC3T3-E1 cells induces proliferation and suppresses differentiation through phosphorylation of ERK1/2. It is therefore suggested that the rupture of balance from differentiation to proliferation induced by RHAMM overexpression may be linked to the pathogenesis of bone neoplasms such as human cementifying fibroma [136].

Importantly, RHAMM has been indicated as a specific target in cancer. Thus, TCR-transgenic lymphocytes specific for RHAMM limit tumor outgrowth *in vivo* in various solid and leukemia tumor models [137]. It has been suggested that immunotherapies like peptide vaccination or adoptive transfer of RHAMM-specific T cells might improve the immune response and the outcome of acute myeloid leukemia patients [138]. Moreover, it has been shown *in vivo* that sulfated hyaluronan augmented tumor growth due to a blockade in complex formation between phosphoinositide 3-kinase (PI3K) and hyaluronan receptors and to a transcriptional downregulation of HA receptors, CD44, and RHAMM, along with PI3K inhibition [139]. In *in vitro* prostate cancer models the antitumor activity of hyaluronan synthesis inhibitor 4-methylumbelliferone was shown to be perpetrated through the downregulation of prostate cancer cells' proliferation, motility, and invasion [140].

Cell surface RHAMM can interact with the second specific hyaluronan receptor [141, 142], CD44, and modulate cell motility, wound healing, and signal transduction. More importantly, cell surface RHAMM can have invasive functions similar to CD44 and can even substitute for CD44 functions [143]. 

## 6. Role of CD44 in Cancer Progression: Focus on Fibrosarcoma

CD44 is a well-characterized transmembrane glycoprotein that has the ability to specifically bind to hyaluronan as well as to participate in the regulation of cell-cell contacts and cell-matrix interface [134, 135]. CD44 binds to hyaluronan through its extracellular domain, whereas its cytoplasmic tail acts as an intracellular signaling pathway activator that is involved in the association of signaling complexes with the actin cytoskeleton [62, 144–146]. The cytoplasmic tail of CD44 interacts with various molecules regulating different signaling pathways [147, 148]. This receptor is encoded by a single gene but can exist in multiple isoforms that are generated both by alternative splicing of its 20 exons and through posttranslational modifications [149]. The altered splice variants expressed in cancer cells generally increase the ability of cancer cells to bind hyaluronan which ultimately results in an induction of tumorigenicity [62]. The most commonly expressed CD44 isoform is the standard CD44s, an 85 kDa protein that contains none of the variable exons. This CD44 isoform acts as a mediator of HA-promoted motility in breast cancer cell lines [58, 114, 150]. Alternative CD44 isoforms that can also bind hyaluronan and transduce its signaling are the so-called variable (v) isoforms [151]. The expression of discrete CD44 splice variants seems to be tumor specific. Thus, the dermatofibroma cells are negative for CD44v3, CD44v4, CD44v6, CD44v7, and CD44v7v8 but show a strong reactivity for CD44v5 and CD44v10. In contrast, CD44s' expression was significantly reduced or absent in all dermatofibrosarcoma protuberans lesions [66].

CD44 appears to be a mediator of fibrosarcoma development and metastatic dissemination. Importantly, CD44 was the only adhesion-related molecule consistently expressed among the early metastatic colonies derived from tumor clones of a murine fibrosarcoma [152]. Thus, hCD44s overexpression and possibly its ability to bind HA are critical for conveying metastatic competence but are antagonistic or selected against during aggressive primary tumor or overt metastasis outgrowth of fibrosarcoma cells [153]. Specifically, overexpression of human CD44s promotes lung colonization during micrometastasis of murine fibrosarcoma cells and facilitates their retention in the lung vasculature [154]. The described plasticity of CD44 gene expression in fibrosarcoma during metastasis could be relevant to discrete metastasis stages [155]. These studies suggest that CD44s may be a critical component of the fibrosarcoma metastatic phenotype induced by specific oncogenes [154]. 

Furthermore, CD44 expressed by HT1080 cells was established to be mainly activated which distinguishes its ability to bind hyaluronan and to mediate downstream signaling [156]. Upon binding to CD44 isoforms, HA initiates tumor cell activities including tumor cell adhesion, growth, survival, migration, invasion, and tumour progression through the activation of intracellular signaling pathways. Specifically, results have revealed that HA-CD44 interactions activate the c-Src kinase, which, in turn, induces twist phosphorylation, leading to the stimulation of miR-10b expression. This sequence of events evokes a reduction of a tumor suppressor protein (HOXD10), upregulates RhoA/RhoC, activates Rho-kinase (ROK), promoting breast tumor cell invasion [157]. 

Regulated uptake of hyaluronan via a CD44 receptor-mediated endocytosis pathway and subsequent degradation by HYAL2 may be important for tumor growth and progression either through the stimulation of angiogenesis or through degradation of HA around blood vessels promoting tumor metastasis [158–160]. Interestingly, CD44 can mediate the adhesion of platelets to hyaluronan secreted by fibrosarcoma cells [161].

Results have indicated that CD44 coimmunoprecipitates and colocalizes with cell surface RHAMM in invasive breast cancer cells, acting together in a HA-dependent autocrine mechanism to regulate signaling through ERK1,2, leading to an increase cell migration [162]. Moreover coexpression of CD44 and RHAMM is associated with poor prognosis in B-cell lymphomas implicating that the interaction of these two proteins may have a clinical significance. These two hyaluronan receptors connect and are involved in many common signaling pathways such as the ones that include VEGF, HGF, HA, Src, ERK1/2, and Fos that regulate cell migration. Other CD44/RHAMM networks that are associated with proliferation, growth, and cancer include MAFG, DYNLL1, MAFK, and FAM83D mediators, which are known to regulate the formation of cell mitotic spindle [163]. It has been proposed that RHAMM and CD44 receptors cooperate in order to induce the cell growth of cementifying fibroma cells. More specifically RHAMM interacts with ERK increasing the proliferative ability of these cancer cells through a mechanism that involves the interaction of CD44 with the epidermal growth factor receptor (EGFR) [164].

## 7. Conclusions

Hyaluronan/RHAMM/CD44 signaling can affect key cellular functions (Figure 1) and is strongly indicated in fibroblastoid cell malignant transformation and concomitant disease progression. Importantly, this signaling mediates fibrosarcoma cell behavior and regulates their specific cell-matrix interactions. Unraveling the complex characteristics of the hyaluronan/RHAMM/CD44 signaling axis in fibrosarcoma may reveal specific targets of pharmacological interventions. 

## Figures and Tables

**Table 1:** Common fibrosarcoma types.

Childhood	Adult
1,5% of childhood malignancies	0,7% of adult malignancies
Spindle shaped malignant cells often interdigitating in a “herringbone” pattern	Spindle shaped malignant cells often interdigitating in a “herringbone” pattern
Less aggressive	More aggressive
Good prognosis	Poor prognosis
Genetic alterations may be involved	Genetic alterations may be involved
